# On Nævi

**Published:** 1824-10

**Authors:** 


### Art. IX.—*On Nævi.*

*To the Editors of the London Medical and Physical Journal.*

Though discredited by most medical men, there is no point of more general popular belief, and especially among females, than that "mother's marks," as they are vulgarly termed, are caused by some sight or longing of the mother during pregnancy. I am acquainted with a lady, the mother of a fine family without a blemish, who is much distressed by a discoloration of this description on the face of her youngest child, and which she believes was produced by going to see some wax-work figures, among which the assassination of Marat, concealed behind a curtain, is calculated to produce great horror in a delicate female: and certainly the discoloration in question has a strong resemblance to the blood sprinkled over the breast of that horrid monster. If so, it is deserving of notice as a physiological fact, that it took place at the very earliest period of gestation.

It would be esteemed a singular obligation, if any your numerous readers can point out a remedy for the defect in question: and it would prove no inconsiderable public benefit if the publication of this letter, by meeting the eyes of those who have power to remedy the evil in question, should tend to diminish the number of disgusting and distressing objects who exhibit themselves in the streets to obtain charity; as I am certain that such sights have harassed and dwelt upon the minds of many nervous, delicate invalids for a considerable time, to the great injury of their health, of both body and mind. Whenever such spectacles of mutilated deformity or disease are proved, by medical certificate, to be unable to maintain themselves otherwise than by begging, there can, I think, be as little question, in a Christian country, of the justice of supporting them at the expense of the State, as of the policy of withdrawing them from the public gaze.

I am, Gentlemen, your most obedient servant,

NÆVUS.*

3d *September*, 1824.

* The writer of the above is an able and respectable physician, well known to the public, as well as to the—EDITORS.

